# Entropy removal of medical diagnostics

**DOI:** 10.1038/s41598-024-51268-4

**Published:** 2024-01-12

**Authors:** Shuhan He, Paul Chong, Byung-Jun Yoon, Pei-Hung Chung, David Chen, Sammer Marzouk, Kameron C. Black, Wilson Sharp, Pedram Safari, Joshua N. Goldstein, Ali S. Raja, Jarone Lee

**Affiliations:** 1 Massachusetts General Hospital and Harvard Medical School, Boston, MA USA; 2 Campbell University School of Osteopathic Medicine, Lillington, NC USA; 3 Department of Electrical and Computer Engineering, Texas A&M University, College Station, TX USA; 4 Brookhaven National Laboratory, Computational Science Initiative, Upton, NY USA; 5 Temerty Faculty of Medicine, University of Toronto, Toronto, ON Canada; 6 Harvard University Department of Chemistry and Chemical Biology, Cambridge, MA USA; 7 Oregon Health and Science University, Portland, OR USA; 8 Massachusetts General Hospital Institute of Health Professions, Boston, MA USA

**Keywords:** Health care, Medical research

## Abstract

Shannon entropy is a core concept in machine learning and information theory, particularly in decision tree modeling. To date, no studies have extensively and quantitatively applied Shannon entropy in a systematic way to quantify the entropy of clinical situations using diagnostic variables (true and false positives and negatives, respectively). Decision tree representations of medical decision-making tools can be generated using diagnostic variables found in literature and entropy removal can be calculated for these tools. This concept of clinical entropy removal has significant potential for further use to bring forth healthcare innovation, such as quantifying the impact of clinical guidelines and value of care and applications to Emergency Medicine scenarios where diagnostic accuracy in a limited time window is paramount. This analysis was done for 623 diagnostic tools and provided unique insights into their utility. For studies that provided detailed data on medical decision-making algorithms, bootstrapped datasets were generated from source data to perform comprehensive machine learning analysis on these algorithms and their constituent steps, which revealed a novel and thorough evaluation of medical diagnostic algorithms.

## Introduction

The use of medical literature to guide clinical practice as part of evidence-based medicine can reduce the number of medical error-related deaths in the US, which is over 98,000 annually, per IOM^1,2^. The assessment of the diagnostic accuracy of medical decision-making aids and tools is an important step towards this goal of improving patient safety and healthcare provision^3,4^. Standard metrics, including sensitivity, specificity, negative predictive value (NPV), and positive predictive value (PPV), measure the predictive utility of medical decision-making tools^5–9^. The sensitivity and specificity of diagnostic tests, such as the chest x-ray for pneumothorax, are well-established for common illnesses. However, with the myriad of conditions and diagnostic tools available, clinicians often face challenges in selecting the most appropriate order of tests for specific, time-sensitive clinical situations.

Shannon entropy is a core concept in machine learning and information theory, particularly in decision tree modeling of data analytics and machine learning^10^. To date, numerous research-based biological and clinical solutions have been developed based on the principle of Shannon entropy, a measure of uncertainty^11–17^, including diagnostic accuracy evaluation. However, no diagnostic metrics that specifically measure the reduction of diagnostic uncertainty, which often leads to decision paralysis and the "shotgun" diagnostic approach^11^, over-testing, delayed diagnosis, and patient harm^11^, have been extensively applied and explored.

Shannon entropy, defined by Eq. (1), offers a solution:

1 $$H\left( x \right) = - \mathop \sum \limits_{i \epsilon x}^{{}} p_{i} \times log_{2} \left( {p_{i} } \right),$$

where $p_{i}$’s denote the probabilities of the possible outcomes of the event, and $p_{i} \times log_{2} \left( {p_{i} } \right)$ is taken to be zero when $p_{i}$ = 0, justified by the fact that the limit of $p_{i} \times log_{2} \left( {p_{i} } \right)$ is zero as $p_{i}$** → **0^+^. Shannon entropy is maximized for a uniform distribution. For *binary* events, in particular, as is the case in this study, the entropy *H(x)* is at its highest when the probabilities $p_{i}$ are exactly 0.5, that is, when there is the most uncertainty, and is at its lowest (zero) when the outcomes are certain, that is, when the outcome probabilities $p_{i}$ are one and zero, respectively. This corresponds to its application in a clinical setting, where the entropy, or uncertainty of a patient with respect to their diagnosis is maximal when they enter the hospital with no testing or diagnostic evaluation. Various diagnostic tools subsequently reduce this clinical uncertainty, ideally to a definitive diagnosis.

In emergency medicine, removal of entropy using testing and imaging tools can clarify the patient's presentation and optimize medical decision-making in time-sensitive settings. Quantifying entropy removal can elucidate the utility and sequence of diagnostic tools in removing uncertainty in those clinical settings and first exclude urgent, lethal pathology. In this study, we aim to characterize the utility and validity of Shannon entropy removal to reanalyze the performance of 623 clinical decision support tools in a publicly available database^18^ compared to traditional validity tools including sensitivity, specificity, PPV/NPV, Youden’s index, and diagnostic odds ratio.

## Materials and methods

### IRB statement

This study is exempt from IRB review of Massachusetts General Hospital and Harvard Medical School as research involves collecting and studying existing data of which sources are publicly available, and subjects cannot be identified directly or through identifiers linked to the subjects.

### Data compilation

**Figure 1:**
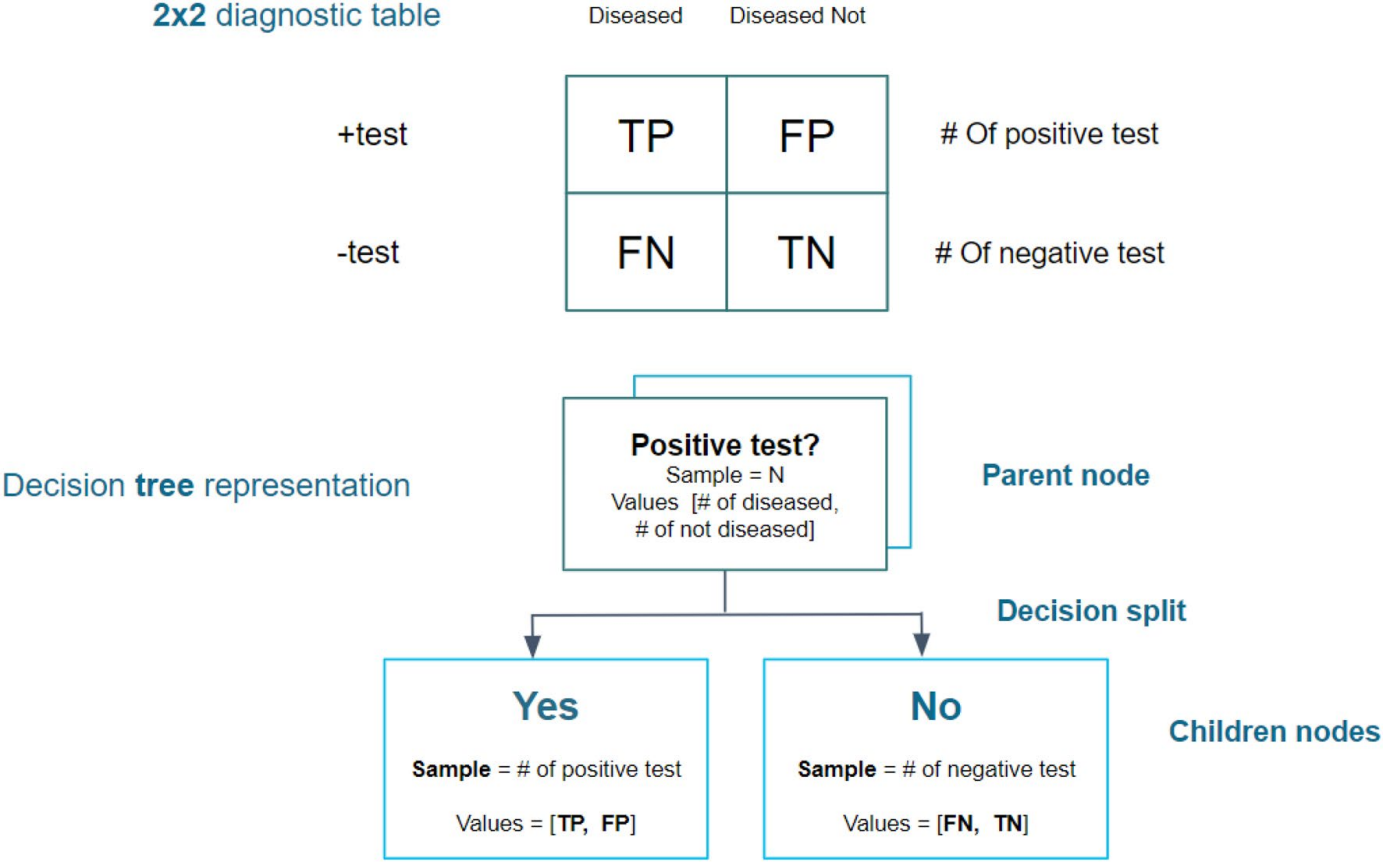
Decision tree representation of 2 × 2 diagnostic table. Diagnostic variables (TP/FP/FN/TN) are utilized to represent a 2 × 2 table and its corresponding medical decision-making tool as a decision tree for entropy analysis.

Diagnostic metrics (true and false positives and negatives, respectively) were compiled from an established online database of diagnostic accuracy, known as “Get the Diagnosis”, totaling 533 studies of 623 decision-making tools of 267 diagnoses^18^. Data collection was performed from November 17, 2022 through January 22, 2023. PubMed was utilized when studies cited from the online database were unable to be accessed directly; concomitant diagnostic tools were also separately explored for elements included in the database as applicable (for example, if studies that evaluated the diagnostic accuracy of mammography for breast cancer screening were included in the database, data was also compiled for low-dose computerized tomography (CT) scans for breast cancer screening; see Data availability statement for details). This data was used to calculate sensitivities, specificities, NPVs, and PPVs. In addition, the data was used to generate decision tree representations for each decision-making tool from which Shannon entropy and entropy removal were calculated (see “Decision tree representation” and Fig. 1 in addition to “Entropy calculation”).

In this study, patient-derived datasets were systematically bootstrapped using the decision tree data previously reported in the literature (see “Machine learning modeling and analysis”). This data was specifically derived from the “Step-By-Step Approach to Febrile Infants” and the “Pediatric Emergency Care Applied Research Network (PECARN) Pediatric Head Injury/Trauma Algorithm”^19–21^.

Similar methods have been performed in other studies to generate health data for the evaluation of healthcare solutions from datasets, such as HES, A&E, and MIMIC^22,23^. In each case, synthetic datasets that preserved the statistical properties of the original real data were generated^24^. This was accomplished by using the decision tree data provided in the original and validation papers of the respective studies and synthesizing a binary dataset of the relevant metrics of each respective algorithm (ex. leukocyturia, age less than 21 days, loss of consciousness, etc.) and their binary value (0 for absence, 1 for presence). Thus, the original data used in the study was recreated with each patient in the respective studies being simplified to only their characteristics relevant to the study in addition to being reduced to a set of binary values for machine learning modeling.

### Decision tree representation

Decision trees are constituted of parent nodes that split to yield children nodes; these nodes and decision splits are able to be generated to produce decision tree representations for diagnostic tools by using the diagnostic metrics for medical decision-making tools from 2 × 2 diagnostic tables as in Fig. 1, where N is the sample size of the study, TP is the number of true positives, FP is the number of false positives, FN is the number of false negatives, and TN is the number of true negatives.

### Entropy calculation

Using diagnostic metrics (N, TP, FP, etc.), Shannon entropy was calculated as below in Eqs. (2) through (4) for the parent node and children nodes, with n_positive_ and n_negative_ representing the number of positive and negative tests, respectively:

2 $$entropy_{parent \,node} = \left[ {\frac{FP + TN}{N} \times \left( {log_{2} \left( N \right) - log_{2} \left( {FP + TN} \right)} \right)} \right] + \left[ {\frac{TP + FN}{N} \times \left( {log_{2} \left( N \right) - log_{2} \left( {TP + FN} \right)} \right)} \right]$$

3 $$entropy_{child \,node \,1} = \left[ {\frac{TP}{{n_{positive} }} \times \left( {log_{2} \left( {n_{positive} } \right) - log_{2} \left( {TP} \right)} \right)} \right] + \left[ {\frac{FP}{{n_{positive} }} \times \left( {log_{2} \left( {n_{positive} } \right) - log_{2} \left( {FP} \right)} \right)} \right]$$

4 $$entropy_{child \,node \,2} = \left[ {\frac{FN}{{n_{negative} }} \times \left( {log_{2} \left( {n_{negative} } \right) - log_{2} \left( {FN} \right)} \right)} \right] + \left[ {\frac{TN}{{n_{negative} }} \times \left( {log_{2} \left( {n_{negative} } \right) - log_{2} \left( {TN} \right)} \right)} \right]$$

Entropy removal was calculated by Eq. (5), where entropy removal equals the difference between the entropy of the parent node (the total entropy of the system) and the weighted average entropy of the children nodes (proportional to n_positive_ and n_negative_, respectively):

5 $$\begin{aligned} & entropy \,removal = \left[ {\left( {\frac{FP + TN}{N} \times (log_{2} \left( N \right) - log_{2} \left( {FP + TN} \right)} \right) + \left( {\frac{TP + FN}{N} \times (log_{2} \left( N \right) - log_{2} \left( {TP + FN} \right)} \right)} \right] \\ & \quad - \left[ {\left[ {\left( {\frac{{n_{positive} }}{N}} \right)\left( {\frac{TP}{{n_{positive} }} \times (log_{2} \left( {n_{positive} } \right) - log_{2} \left( {TP} \right))} \right) + \left( {\frac{FP}{{n_{positive} }} \times (log_{2} \left( {n_{positive} } \right) - log_{2} \left( {FP} \right))} \right)} \right]} \right. \\ & \quad + \left. {\left[ {\left( {\frac{{n_{negative} }}{N}} \right)\left( {\frac{FN}{{n_{negative} }} \times (log_{2} \left( {n_{negative} } \right) - log_{2} \left( {FN} \right)} \right) + \left( {\frac{TN}{{n_{negative} }} \times (log_{2} \left( {n_{negative} } \right) - log_{2} \left( {TN} \right)} \right)} \right]} \right] \\ \end{aligned}$$

Data provided in the validation study by Gomez et al. was utilized to generate a patient dataset for analysis of the Step-By-Step Approach to Febrile Infants^19^. Data provided in the original study by Kupperman et al. was similarly utilized to generate two separate patient datasets for analysis of the PECARN Pediatric Head Injury/Trauma Algorithm: one for patients less than 2 years of age and another for patients greater than or equal to 2 years of age^20^.

### Machine learning modeling and analysis

The Python MATLAB and scikit-learn packages were utilized in this study to generate, analyze the performance, and visualize machine learning models developed from the synthetic patient datasets (for more details regarding the machine learning models developed)^25,26^. Decision tree-based diagnostic algorithms pose unique applications for Shannon entropy analysis of the decision-making tool in its entirety and its constituent steps/nodes, allowing for evaluation of each feature in the algorithm. A decision tree was produced for each patient dataset and these decision trees were subsequently analyzed for the entropy removal and feature importance of each step within the algorithm. In the context of machine learning, feature importance is defined as the relative importance of each feature when making a prediction and is calculated as the decrease in entropy weighted by the probability of reaching that node, as shown below in Eqs. (6) and (7):

6 $$ni_{j} = w_{j} C_{j} - w_{left\left( j \right)} C_{left\left( j \right)} - w_{right\left( j \right)} C_{right\left( j \right)}$$

(ni_j_ = the importance of node j, w_j_ = weighted number of samples reaching node j, C_j_ = the impurity value of node j, left(j) = child node from left split on node j, right(j) = child node from right split on node j).

7 $$fi_{i} = \frac{{\mathop \sum \nolimits_{j: \,node \,j \,splits \,on \,feature i}^{{}} ni_{j} }}{{\mathop \sum \nolimits_{k\epsilon \,all \,nodes}^{{}} ni_{k} }}$$

(fi_i_ = the importance of feature I, ni_j_ = the importance of node j, ni_k_ = the importance of node k).

## Results

### Entropy removals

Entropy removal was calculated in addition to sensitivity, specificity, NPV, and PPV as well as diagnostic odds ratio and Youden’s index for 533 studies to evaluate the 623 medical decision-making tools.

**Figure 2:**
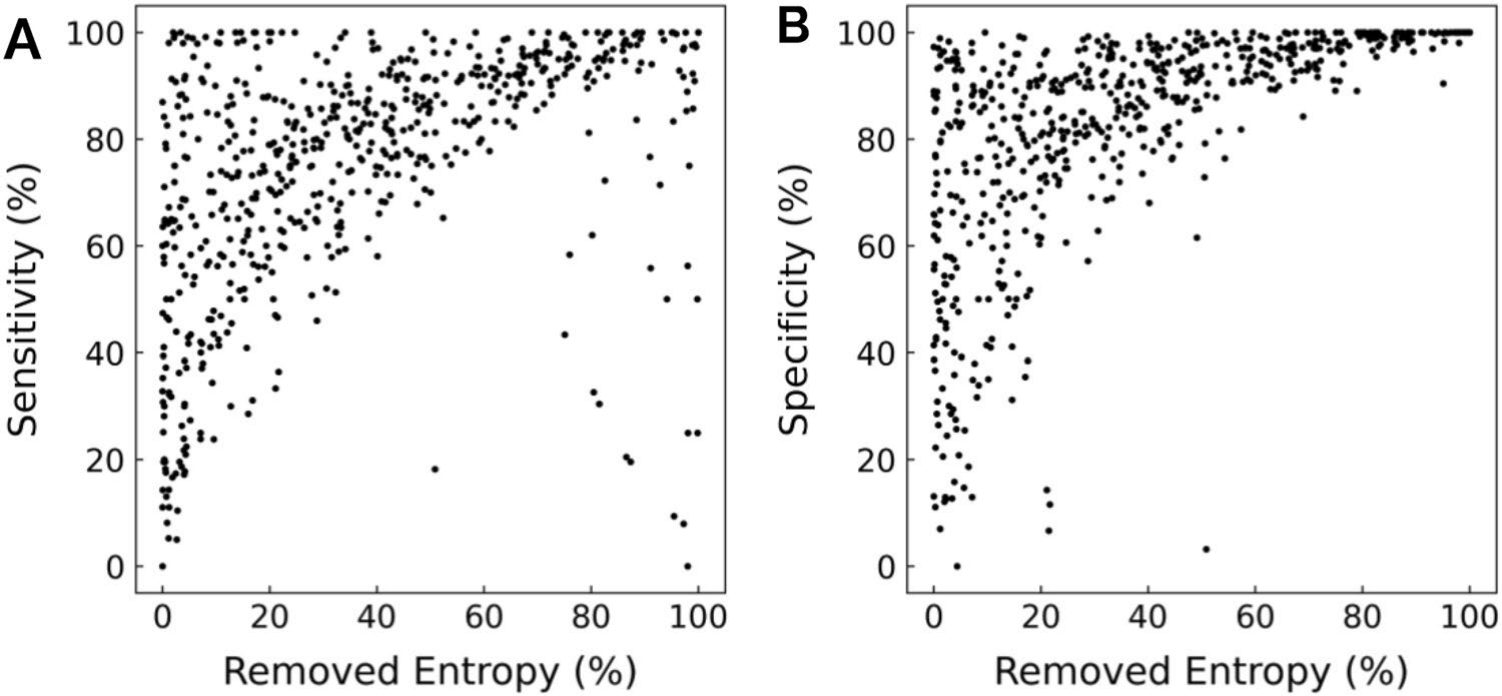
Scatterplot of removed entropy and tool sensitivity and specificity. 623 medical decision-making tools were analyzed. (**A**) Sensitivity exhibits a 0.46 Pearson correlation and 0.55 Spearman correlation with entropy removal (*p* < .001). (**B**) Specificity exhibits a 0.61 Pearson correlation and 0.74 Spearman correlation with entropy removal (*p* < .001).

**Figure 3:**
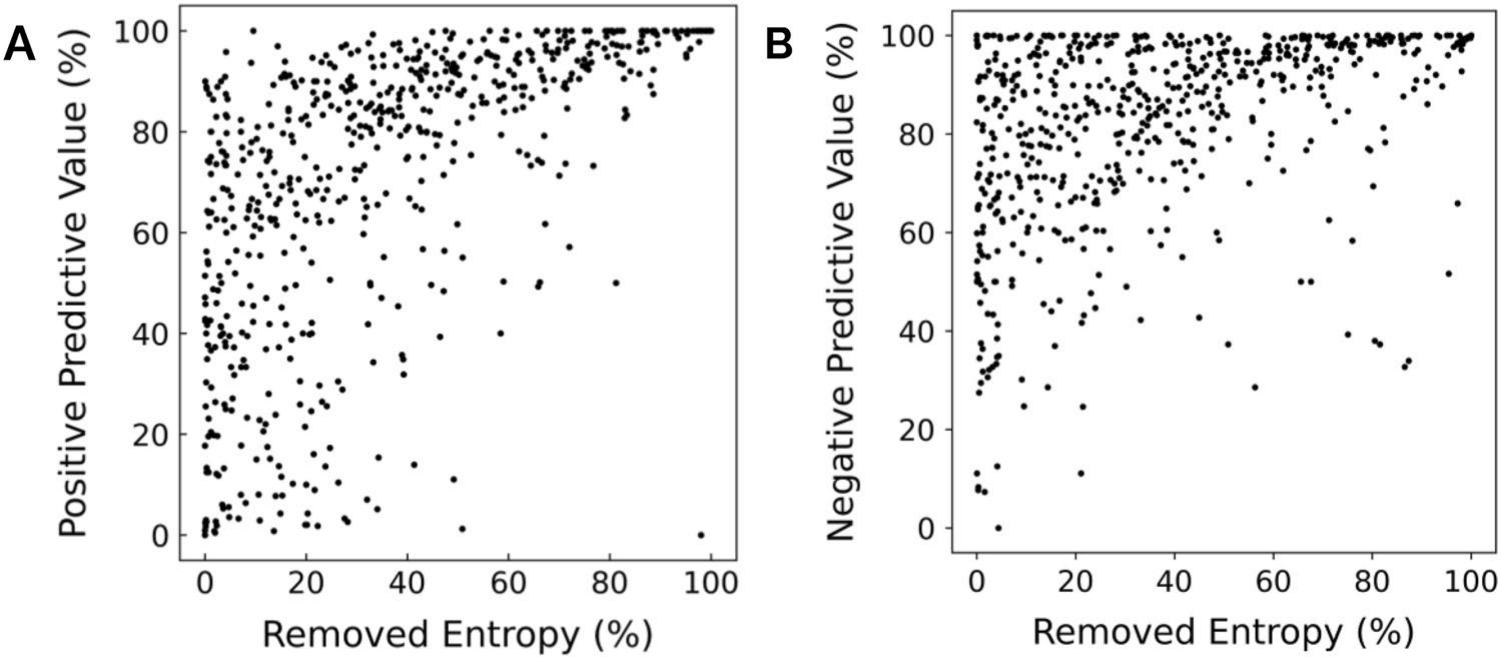
Scatterplot of removed entropy and tool positive predictive value and negative predictive value. 623 medical decision-making tools were analyzed. (**A**) Positive predictive value exhibits a 0.60 Pearson correlation and 0.71 Spearman correlation with entropy removal (*p* < .001). (**B**) Negative predictive value exhibits a 0.41 Pearson correlation and 0.46 Spearman correlation with entropy removal (*p* < .001).

Entropy removal displayed significant but weak positive correlations with sensitivity and NPV and showed significant moderate positive correlations with specificity and PPV (*p* < 0.001). Entropy removal exhibited significant strong positive correlations with comprehensive clinical diagnostic metrics, such as Youden’s index and logged diagnostic odds ratio (*p* < 0.001). Z-score calculation for differences in correlations revealed significant differences between the respective correlations of Youden’s index and logged diagnostic odds ratio with entropy removal as compared to the correlations of the other explored diagnostic metrics with entropy removal (*p* < 0.001). Figures 2 and 3 illustrate the correlation between different diagnostic metrics and entropy removal. Tables 1 and 2 provide the Pearson and Spearman correlation coefficients for entropy removal and the different diagnostic metrics, respectively. Tables 3 and 4 provide examples of comparisons of the different diagnostic accuracy metrics of tests evaluating for pneumothorax and thoracic aortic dissection, respectively. Table 5 displays the results of entropy removal analysis of decision tree-based clinical algorithms and their constituent steps.

**Table 1:** Pearson correlation coefficients of diagnostic metrics and entropy removal.

Metric	Pearson coefficient
Sensitivity	0.465
Specificity	0.607
PPV	0.600
NPV	0.407
Logged diagnostic odds ratio	0.909
Youden’s index	0.780

623 diagnostic tools were analyzed (*p* < .001). *Note*: diagnostic odds ratio was logged for correlation analysis because it displayed an exponential relationship with entropy removal.

**Table 2:** Spearman correlation coefficients of diagnostic metrics and entropy removal.

Metric	Spearman coefficient
Sensitivity	0.550
Specificity	0.741
PPV	0.712
NPV	0.456
Logged diagnostic odds ratio	0.945
Youden’s index	0.890

623 diagnostic tools were analyzed (*p* < .001). *Note*: diagnostic odds ratio was logged for correlation analysis because it displayed an exponential relationship with entropy removal.

**Table 3:** Comparison of diagnostic metrics of tests for pneumothorax.

Pneumothorax evaluation	Entropy removal	Sensitivity (%)	Specificity (%)	NPV (%)	PPV (%)
Chest US	82.38%	95.12	98.87	96.89	98.20
Supine AP CXR	40.94%	55.83	100	100	86.02

Chest ultrasound (US) for pneumothorax diagnosis showed greater entropy removal than supine anterior–posterior (AP) chest x-ray (CXR).

**Table 4:** Comparison of diagnostic metrics for thoracic aortic dissection evaluation.

Thoracic aortic dissection evaluation	Entropy removal (%)	Sensitivity (%)	Specificity (%)	NPV (%)	PPV (%)
TEE	83.69	94.80	99.28	98.76	96.92
Helical CT	87.23	97.64	98.90	99.31	96.28
MRI	81.36	93.33	99.30	98.32	97.13

Helical CT scan had the greatest entropy removal for thoracic aortic dissection when compared with transesophageal echocardiogram (TEE) and magnetic resonance imaging (MRI).

**Table 5:** Results of machine learning analysis for medical decision-making algorithms.

MDM algorithm	ML model prediction accuracy	Entropy removal (percentage)	Most important metric (as defined by entropy removal)	Most important metric (as defined by feature importance)
Step-by-step approach to febrile infants	0.963	0.0295 (11.9%)	Abnormal pediatric triangle assessment/ill-appearing (0.0117)	Abnormal pediatric triangle assessment/ill-appearing (0.395)
PECARN (age < 2 years)	0.994	0.0130 (16.5%)	Altered mental status (0.00783)	Altered mental status (0.302)
PECARN (age ≥ 2 years)	0.990	0.0111 (16.8%)	Altered mental status (0.00729)	Altered mental status (0.655)

Three medical decision-making algorithms were analyzed using bootstrapped data, revealing their robust diagnostic value and providing in-depth insight on each algorithm’s individual steps. PECARN: Pediatric Emergency Care Applied Research Network Pediatric Head Injury/Trauma Algorithm.

The diagnostic metrics of different diagnostic tools that assess patients for the same pathology were able to be compared as in Tables 3 and 4.

### Bootstrapping and stepwise entropy calculation

Decision tree machine learning analysis of the generated patient datasets yielded the exact decision trees of the original algorithms, supporting the validity of the clinical algorithms. Table 5 shows the results of machine learning analysis.

## Discussion

Our study demonstrates the potential utility of quantified entropy removal of medical diagnostic decision-making tools. Diagnostic tools that are 100% sensitive and 100% specific (or definitively diagnostic) also have an entropy removal of 100% as all entropy (uncertainty) is completely removed with regard to a particular pathology. In cases in which diagnostic tools are less than 100% sensitive and/or specific, our entropy removal calculations provide further insight into how much diagnostic value the tool provides. In other words, entropy removal may be used as a “meta-metric” to assess existing clinical diagnostic metrics. The strong positive correlations of entropy removal with established comprehensive measures of diagnostic accuracy (Youden’s index and logged diagnostic odds ratio) may support its validity while its distinctive advantages support its novelty. Entropy removal provided unique insight on the diagnostic value of medical decision-making tools beyond the limitations of Youden’s index and diagnostic odds ratio (which include the omission of disease prevalence in calculation as well as inherent limitations of calculation in the respective formulas), demonstrating its clinical utility with particular potential in the setting of Emergency Medicine where exclusion of critical diagnoses within time-limited emergencies is critical. This utility of entropy removal in assessment of the diagnostic value of medical decision-making tools can be also seen in comparing different tools that evaluate for the same pathology.

Traditional measures of test quality, such as sensitivity and specificity, are not as easily used for comparing diagnostic strategies as entropy removal. For example, evaluation for thoracic aortic dissection via helical CT scan has a sensitivity of 97.64% and a specificity of 98.90%, whereas evaluation by way of MRI has a slightly lower sensitivity (93.33%) but a higher specificity (99.30%). Entropy removal calculation reveals that helical CT scan removes 87.23% of all entropy with respect to thoracic aortic dissection while MRI removes 81.36%, revealing the superior overall diagnostic value of a helical CT scan in assessment for thoracic aortic dissection. This demonstrates the ability of entropy removal to provide clarification and stratification that sensitivity and specificity do not offer. This advantage of entropy removal calculation can also be seen in the comparison between chest x-ray and low-dose CT scan for lung cancer screening, with CXR having a greater sensitivity (88.89% versus 73.38%) and low-dose CT having a greater specificity (92.60% versus 97.00%) but CXR having greater entropy removal (32.03% versus 28.20%).The superior imaging test for pneumothorax can also be identified by entropy removal calculations, as chest ultrasound yields a superior sensitivity (95.12% versus 55.83%) while supine AP chest x-ray provides a greater specificity (98.87% versus 100%), but chest ultrasound has greater entropy removal over supine AP chest x-ray (82.38% versus 40.94%). Entropy removal thus has the potential to provide an evidence-based foundation for the dynamic evaluation of patients, as it can potentially serve as the basis for guiding medical decision-making in the context of performing certain tests or utilizing particular tools in time restricted order to most effectively eliminate uncertainty regarding a patient’s acute care presentation.

Quantifying the entropy removal capability of medical diagnostics also opens the door for further exploration in healthcare innovation, such as the quantification of the impact of clinical guidelines by analyzing and comparing the diagnostic value of decision-making tools and tests. Entropy removal calculation also has potential use in financial analysis of healthcare costs, as metrics such as entropy removal per cost could be calculated and used to evaluate healthcare cost efficiency. For example, metrics such as the percent entropy removed per US dollar (USD) by a diagnostic tool can be calculated. Using publicly available Medicare costs^27^, a chest x-ray screening for lung cancer was found to remove 1.28% entropy per USD while a low-dose CT scan screening for lung cancer removed 0.27% entropy per USD. Similarly, a chest US evaluating for pneumothorax removes 3.30% entropy per USD while a CXR evaluating for pneumothorax removes 1.64% entropy per USD. As a final example, an US of the abdomen evaluating for nephrolithiasis removes 0.13% entropy per USD and a CT scan of the abdomen evaluating for nephrolithiasis removes 0.11% entropy per USD.

With respect to entropy removal, in the examples above, it would be more cost-effective to pursue chest x-ray imaging to screen for lung cancer screening as well as to evaluate for pneumothorax as opposed to low-dose CT and ultrasound, respectively. With regard to nephrolithiasis assessment, a CT scan removes marginally more entropy than ultrasound, but has inferior cost-effectiveness (as measured by entropy removal per USD) compared to ultrasound. All these results have the potential to inform medical decision-making in various contexts, providing an alternative means of cost-effectiveness analysis in assessing the efficiency of healthcare systems.

Furthermore, entropy removal can be used to evaluate the diagnostic quality of entire departments or systems. For example, the diagnostic performances of expert radiologists and residents regarding COVID-19 identification on chest x-rays was evaluated in a 2021 study, which found that attending radiologists diagnosed COVID-19 at a sensitivity of 78.98% and a specificity of 80.45% as opposed to resident radiologists (75.09% and 57.89%, respectively)^28^. Entropy removal calculations can be used to further evaluate the diagnostic quality of each respective subgroup, revealing that attending radiologists removed 24.55% of clinical uncertainty regarding COVID-19 via chest x-rays while residents only removed 7.55%.

Shannon entropy, proposed as a big data metric^29^, can evaluate diagnostic quality across entire hospitals or health networks, not just specific pathologies. The utility of Shannon entropy can be extended to other research applications where data points of true and false positives and negatives are reported. Beyond individual groups, entropy removal can gauge the performance of whole departments and networks, indicating healthcare innovation and quality. Additionally, it can highlight healthcare disparities by comparing diagnostic efficiency across various regions and patient groups.

The generation of synthetic patient datasets from medical decision-making algorithms and subsequent analysis of these algorithms by decision tree machine learning analysis as performed in this study showed potential utility, as well, though with limitations (see limitations below). The resultant machine learning decision trees and calculated metrics from the algorithms evaluated in this study were in line with the medical decision-making algorithms used in practice and the results of this analysis can be understood to support and further validate these current clinical guidelines. The results also quantified the effectiveness of the individual constituent steps of the algorithms, providing measurable insight on the most clinically relevant information for patient assessment in the algorithms. If more data are provided in literature for the development and validation of medical decision-making algorithms, deeper analysis can be performed on these diagnostic tools in order to more thoroughly evaluate them.

The limitations of this study include the fact that the findings outlined in this study are statistical and mathematical modeling that will require further application to clinical practice. While the application of Shannon entropy to medical diagnostics, as in this study, is a limited implementation of established information theory and machine learning concepts to publicly available data, the need for clinical validation still remains. For example, the presence of differences in entropy removal from established metrics does not necessarily establish that such differences are clinically meaningful or accurately reflect the performance of the decision support tools unless some prospective testing is done. While this was outside of the scope of this study, further investigation and validation exploring these phenomena is warranted. Furthermore, the stepwise evaluation of algorithms as described in the latter portions of this paper made use of bootstrapped (resampled) data, which is very internally consistent but also requires prospective and external validation. Larger data sets from healthcare electronic medical records could provide valuable insight using our entropic approach.

## Data Availability

All data (including the specific studies and figures utilized in compiling diagnostic variables) have been uploaded into a public repository which can be accessed at the following URL: https://data.mendeley.com/datasets/hgwdb4mtpw/2 3.0^30^.

## References

[1] Guyatt G (1992). Evidence-based medicine: A new approach to teaching the practice of medicine. JAMA.

[2] Kohn KT, Corrigan JM, Donaldson MS (1999). To Err Is Human: Building a Safer Health System.

[3] “AHRQ National Scorecard on Hospital-Acquired Conditions Updated Baseline Rates and Preliminary Results 2014–2017” (Agency for Healthcare Research and Quality, 2019).

[4] Newman TB, Kohn MA (2020). Evidence-Based Diagnosis: An Introduction to Clinical Epidemiology.

[5] Bartol T (2015). Thoughtful use of diagnostic testing: Making practical sense of sensitivity, specificity, and predictive value. Nurse Practit..

[6] Naeger DM, Kohi MP, Webb EM, Phelps A, Ordovas KG, Newman TB (2013). Correctly using sensitivity, specificity, and predictive values in clinical practice: How to avoid three common pitfalls. Am. J. Roentgenol..

[7] Eusebi P (2013). Diagnostic accuracy measures. Cerebrovasc. Dis..

[8] Parikh R, Mathai A, Parikh S, Chandra Sekhar G, Thomas R (2008). Understanding and using sensitivity, specificity and predictive values. Indian J. Ophthalmol..

[9] Monaghan TF, Rahman SN, Agudelo CW (2021). Foundational statistical principles in medical research: Sensitivity, specificity, positive predictive value, and negative predictive value. Medicina.

[10] Casagrande A, Fabris F, Girometti R (2022). Fifty years of Shannon information theory in assessing the accuracy and agreement of diagnostic tests. Med. Biol. Eng. Comput..

[11] Ehrmann DE, Joshi S, Goodfellow SD (2023). Making machine learning matter to clinicians: model actionability in medical decision-making. NPJ Digit. Med..

[12] Lotfi FH, Fallahnejad R (2010). Imprecise Shannon's entropy and multi attribute decision making. Entropy.

[13] Ting HW, Wu JT, Chan CL, Lin SL, Chen MH (2010). Decision model for acute appendicitis treatment with decision tree technology—A modification of the Alvarado scoring system. J. Chin. Med. Assoc..

[14] Bertolini S, Maoli A, Rauch G, Giacomini M (2013). Entropy-driven decision tree building for decision support in gastroenterology. Stud. Health Technol. Inform..

[15] Liu Y, Jiao Y, Fan Q (2021). Shannon entropy for time-varying persistence of cell migration. Biophys. J..

[16] Halma MTJ, Ritchie DB, Woodside MT (2021). Conformational shannon entropy of mRNA structures from force spectroscopy measurements predicts the efficiency of -1 programmed ribosomal frameshift stimulation. Phys. Rev. Lett..

[17] Monaco A, Amoroso N, Bellantuono L (2019). Shannon entropy approach reveals relevant genes in Alzheimer's disease. PLoS ONE.

[18] Hammer MM (2017). Kohlberg GDGet the diagnosis: An evidence-based medicine collaborative Wiki for diagnostic test accuracy. Postgrad. Med. J..

[19] Gomez B, Mintegi S, Bressan S (2016). Validation of the “step-by-step” approach in the management of young febrile infants. Pediatrics.

[20] Kuppermann N, Holmes JF, Dayan PS (2009). Identification of children at very low risk of clinically-important brain injuries after head trauma: A prospective cohort study. Lancet.

[21] Berrar D, Dubitzky W, Dubitzky W, Wolkenhauer O, Cho KH, Yokota H (2013). Bootstrapping. Encyclopedia of Systems Biology.

[22] Arvanitis TN, White S, Harrison S, Chaplin R, Despotou G (2022). A method for machine learning generation of realistic synthetic datasets for validating healthcare applications. Health Inform. J..

[23] El Emam K, Mosquera L, Fang X, El-Hussuna A (2022). Utility metrics for evaluating synthetic health data generation methods: Validation study. JMIR Med. Inform..

[24] Goncalves A, Ray P, Soper B, Stevens J, Coyle L, Sales AP (2020). Generation and evaluation of synthetic patient data. BMC Med. Res. Methodol..

[25] MATLAB 8.0 and Statistics Toolbox 8.1, The MathWorks, Inc., Natick, Massachusetts, United States.

[26] Pedregosa F (2011). Scikit-learn: Machine learning in python. JMLR.

[27] Procedure Price Lookup for Outpatient Services | Medicare.gov. www.medicare.gov. https://www.medicare.gov/procedure-price-lookup/.

[28] Flor N, Saggiante L, Savoldi AP (2021). Diagnostic performance of chest radiography in high COVID-19 prevalence setting: Experience from a European reference hospital. Emerg. Radiol..

[29] Juszczuk P, Kozak J, Dziczkowski G, Głowania S, Jach T, Probierz B (2021). Real-world data difficulty estimation with the use of entropy. Entropy.

[30] Chong, P. Entropy removal of medical diagnostics. Mendeley Data, V1. 10.17632/hgwdb4mtpw.1 (2023). https://data.mendeley.com/datasets/hgwdb4mtpw/2.

